# How leadership attention is associated with online–offline healthcare service synergy: differentiated effects of noticing, interpreting, and acting

**DOI:** 10.3389/fpubh.2026.1956639

**Published:** 2026-09-29

**Authors:** Zhangzhang Zhao, Wanyu Xu, Yitian Sun, Zehong Zhang

**Affiliations:** School of Medical Humanities and Management, Wenzhou Medical University, Wenzhou, China

**Keywords:** digital health, exploratory sequential mixed-methods, leadership attention, online-offline healthcare service synergy, structural equation modeling

## Abstract

**Aim:**

This study examines how three types of leadership attention (noticing, interpreting, acting) are associated with online-offline healthcare service synergy (process and organizational synergy), along with motivational antecedents (credit claiming, blame avoidance) and contextual boundary conditions, using the APCC framework in the Chinese context.

**Methods:**

An exploratory sequential design was adopted. During the qualitative phase, data were triangulated from 41 interviews (hospital administrators, health bureau officials, clinicians), “Internet + Healthcare” policy documents, and literature review; items were generated via thematic analysis and expert panel validation. For the quantitative phase, quota sampling following regional distribution of China’s internet healthcare users, yielding 450 valid responses from experienced professionals; data were analyzed with SPSS/AMOS (SEM) and PROCESS macro for moderation.

**Results:**

The three attention types showed differentiated patterns. Acting had the strongest positive associations with process synergy (*β* = 0.53, *p* < 0.001) and organizational synergy (*β* = 0.50, *p* < 0.001). Noticing was positively associated with both (*β* = 0.26 and *β* = 0.29, *p* < 0.001). Interpreting was only significantly associated with process synergy (*β* = 0.15, *p* < 0.05), not organizational synergy. Credit claiming positively predicted all three attention types (*β* = 0.78–0.87, *p* < 0.001); blame avoidance negatively predicted them (*β* = −0.46 to −0.39, *p* < 0.001). Contextual factors positively moderated the attention–organizational synergy link (*β* = 0.113, *p* < 0.01, 95%CI [0.02, 0.14]), with no moderation for process synergy.

**Conclusion:**

This study validates the APCC framework for digital healthcare integration, supporting the cognitive-behavioral logic of leadership attention from noticing through interpreting to acting. The findings indicate that only action-oriented attention is associated with addressing structural barriers and achieving organizational synergy, while interpretive attention is only associated with improved technical process integration. The study extends the Attention-Based View to healthcare and provides actionable strategies for advancing online-offline synergy.

## Introduction

Before proceeding to the full analysis, it is necessary to clearly define the two focal constructs of this study.

Online-offline healthcare service synergy refers to overall performance improvement generated by the nonlinear complementary interaction between online and offline healthcare services. Grounded in synergetics theory (1), this construct encompasses two sub-dimensions: process synergy and organizational synergy. Process synergy focuses on patient-centered service chain optimization at the operational level, while organizational synergy prioritizes cross-institutional collaboration at the governance level.

Leadership attention refers to the differential allocation of limited cognitive, temporal, and organizational resources by leaders across domains such as healthcare policy agendas and institutional coordination tasks. Building on Ocasio’s Attention-Based View (ABV) (2), leadership attention is not a unidimensional construct; instead, it comprises three interrelated core dimensions: noticing dimension, interpreting dimension and acting dimension. Ocasio emphasizes that an organization is essentially a “distributed attention system”, whose behaviors can be understood as the outcome of channeling decision-makers’ attention toward specific issues and procedures through rules, resources, and relational structures. In the context of online-offline healthcare integration in this study, this construct extends to digital leadership: rather than remaining at the level of passive attentional awareness, effective leaders must possess corresponding digital literacy and actively guide digital transformation. They identify and judge digital healthcare trends, clarify integration pathways and value orientations, and promote the implementation of resource allocation and institutional adjustments, so as to translate attentional resources into tangible outcomes of online-offline service synergy (3, 4).

The rapid advancement of digital technologies is profoundly reshaping the global healthcare system and accelerating the worldwide diffusion of internet-based healthcare services (5). During the COVID-19 pandemic, online healthcare services—including remote consultation, electronic prescribing, and continuous health management—effectively broke the temporal and spatial constraints of traditional offline care delivery. Their convenience and accessibility not only sustained healthcare service continuity during public health emergencies, but also improved the overall efficiency of routine healthcare provision (6).

However, online healthcare has inherent limitations: diagnostic accuracy remains controversial due to the absence of physical examinations and laboratory testing, and risks of medical data insecurity and patient privacy breaches have escalated (7, 8). In contrast, supported by specialized medical equipment, face-to-face clinical consultations, and well-established quality control systems, offline healthcare holds irreplaceable strengths in diagnostic precision, therapeutic efficacy, doctor-patient trust building, and privacy protection. Multiple studies have confirmed that the convenience of online healthcare and the reliability of offline healthcare are inherently complementary, and their deep integration has become a core evolutionary trend of modern healthcare systems (9). The integrated model can improve chronic disease control outcomes, reduce avoidable hospital readmissions (10), enhance care continuity across healthcare institutions, and narrow the geographic accessibility gap for rural and underserved populations, thereby advancing greater health equity (11, 12).

Existing scholarly literature has advanced research on online-offline healthcare integration from technological, operational, and resource allocation perspectives. Nevertheless, notable research gaps remain. First, most studies conceptualize online-offline healthcare service synergy as a technology- or process-driven outcome, while largely overlooking the organizational and behavioral underpinnings that determine the effective implementation of technological solutions (13). In fact, online-offline healthcare integration inherently involves cross-departmental coordination, resource reallocation, and institutional restructuring, and its effectiveness is largely shaped by how organizational decision-makers formulate strategic priorities. Second, the role of leadership attention—defined as the allocation of leaders’ limited cognitive and decision-making resources to specific issue domains—remains largely underexplored in this research area (14). Grounded in the Attention-Based View (ABV), attention constitutes a scarce strategic resource that shapes an organization’s action priorities and patterns of resource allocation (2). Neglecting variations in leadership attentional focus makes it difficult to account for why similar digital tools and policy mandates yield heterogeneous integration outcomes across healthcare institutions. Third, existing studies have rarely systematically unpacked the motivational antecedents and contextual boundary conditions of leadership-driven integration, particularly in non-Western institutional contexts (15).

The aforementioned research gaps are particularly pronounced in China’s healthcare system, which operates under a pressure-driven governance framework. Its administrative hierarchy functions through top-down target decomposition, performance evaluation, and promotion-linked accountability mechanisms (16). Within this system, local health administrators and hospital leaders face strong incentives and constraints that compel them to prioritize policy mandates from higher authorities. Their allocation of attentional resources is profoundly shaped by the dual logics of pursuing performance rewards and avoiding accountability risks (17). However, how different forms of leadership attention are associated with online-offline healthcare service synergy, and through what specific mechanisms attention allocation is shaped, have yet to be thoroughly examined.

This study employs the comprehensive Antecedent-Process-Context-Consequence (APCC) analytical framework proposed by Xie and Han (18) in Management World. Built upon the meta-theoretical framework of the Attention-Based View (ABV) and the antecedent-consequence theoretical framework (19), this model incorporates process mechanisms and contextual mechanisms to form a complete four-module analytical system. Its core logic is structured as follows: motivational antecedents shape leaders’ attentional allocation preferences; distinct attention configurations are associated with divergent organizational change outcomes; and contextual factors moderate the translation process from attention allocation to outcomes. Empirical applications of this framework have covered multiple research domains, including public policy implementation and public service innovation (18, 20, 21).

As the core focus of this study, online-offline healthcare service synergy follows an inherent behavioral logic of “motivational stimuli – attentional processing – synergy performance”, and is simultaneously influenced by multiple contextual factors across technological, policy, and market dimensions. These attributes make it highly compatible with the analytical structure of the APCC framework. Grounded in this framework, the present study can systematically address three core research questions: how motives shape leadership attention allocation, how different types of attention exert differential effects on synergy performance, and how contextual factors moderate the translation effect of attention into synergy outcomes.

The following is a detailed explanation of the framework:

### Antecedent module

This module focuses on the motivational antecedents of distinct types of leadership attention allocation, addressing why leaders pay attention to online-offline service integration. Drawing on the blame avoidance–credit claiming framework in public administration (22), this study operationalizes key motivational factors into two core logics of leadership behavior:

Blame avoidance refers to leaders’ tendency to evade accountability risks and political costs arising from failed implementation of healthcare integration policies.

Credit claiming refers to leaders’ willingness to gain political recognition and professional reputation by promoting innovation and effective implementation of online-offline healthcare integration.

We hypothesize that these two motives are differentially associated with leaders’ three forms of attention allocation—Noticing, Interpreting, and Acting—thus forming the logical starting point for understanding associations between attention and the process of online-offline healthcare service synergy.

### Process module

This module centers on the mechanism of different types of leadership attention, addressing how leaders are associated with online-offline service synergy via distinct attention configurations. Based on the Attention-Based View (2) and related extensions, this study categorizes healthcare leadership attention into three conceptually distinct types:

Noticing refers to health leaders’ conscious awareness and sustained focus on the agenda of online-offline healthcare integration (23).

Interpreting involves leaders’ systematic construction and clear articulation of the strategic goals, implementation pathways, and core values of online-offline integration (24).

Acting reflects the translation of leadership attention into actionable and substantive measures, such as allocating dedicated resources, adjusting institutional arrangements, and designing incentive schemes for online-offline healthcare integration (14).

These three forms of attention are not simply sequential but coexist and interact, jointly constituting the core mechanism underlying online-offline healthcare service synergy.

### Consequence module

This module defines the performance outcomes of online-offline healthcare service synergy, which serves as the core dependent variable. Grounded in synergetics (1), which emphasizes that sub-systems generate a “1 + 1 > 2” holistic effect through nonlinear interactions, this study classifies synergy performance into two dimensions according to healthcare service characteristics:

Organizational synergy refers to improvements in overall operational efficiency achieved when online healthcare service entities (e.g., internet hospitals, online diagnosis and treatment platforms) and offline healthcare institutions break down organizational boundary barriers through the restructuring of governance rules, alignment of authority and responsibility systems, and resource sharing. At its core, this dimension addresses the challenge of cross-entity collaboration between online and offline stakeholders.

Process synergy refers to enhancements in service efficiency and patient experience delivered through the seamless integration of online and offline service links and end-to-end process optimization across the full patient care journey. Its core purpose is to resolve the fragmentation of service delivery segments. Typical scenarios include the linkage between online appointment registration and offline priority consultation, cross-platform sharing of electronic health records, seamless referral from online triage to offline examinations and laboratory testing, and closed-loop prescription flow from online diagnosis to offline pharmacy pickup (25). For instance, in chronic disease management pathways, patients complete follow-up consultations online, receive coordinated scheduling for offline examinations and laboratory tests, and then access test results and medication guidance via the online platform. This complete closed-loop service pathway represents a concrete manifestation of process synergy (26).

We hypothesize that leaders’ Noticing, Interpreting, and Acting are key correlates of these two types of synergy performance.

### Context module

This module identifies the boundary conditions under which different types of leadership attention translate into online-offline synergy performance. The effectiveness of leadership attention in driving organizational change is inevitably constrained and moderated by multiple internal and external environmental factors (27). Integrating the practical context of China’s healthcare system reform, this study identifies five critical contextual factors: maturity of medical technology infrastructure, intensity of public demand for integrated care, acceptance of healthcare staff toward online-offline service transformation, support from superior health policies for digital healthcare integration, and competitiveness of the local healthcare market (28–30). These contextual factors are expected to moderate the relationships between distinct forms of leadership attention and online-offline synergy performance.

Based on the above theoretical analysis and the tailored APCC framework for China’s healthcare context, this study proposes the following specific research hypotheses:

*H1a–c*: Blame avoidance motive is negatively associated with the noticing (H1a), interpreting (H1b), and acting (H1c) types of leadership attention.

*H2a–c*: Credit claiming motive is positively associated with the noticing (H2a), interpreting (H2b), and acting (H2c) types of leadership attention.

*H3a–b*: The noticing dimension of healthcare leadership attention is positively associated with process synergy (H3a) and organizational synergy (H3b).

*H4a–b*: The interpreting dimension of healthcare leadership attention is positively associated with process synergy (H4a) and organizational synergy (H4b).

*H5a–b*: The acting dimension of healthcare leadership attention is positively associated with process synergy (H5a) and organizational synergy (H5b).

*H6a–b*: Contextual factors positively moderate the relationship between healthcare leadership attention and process synergy (H6a) as well as organizational synergy (H6b).

The conceptual framework of the hypothesized model is presented in Figure 1.

**Figure 1:**
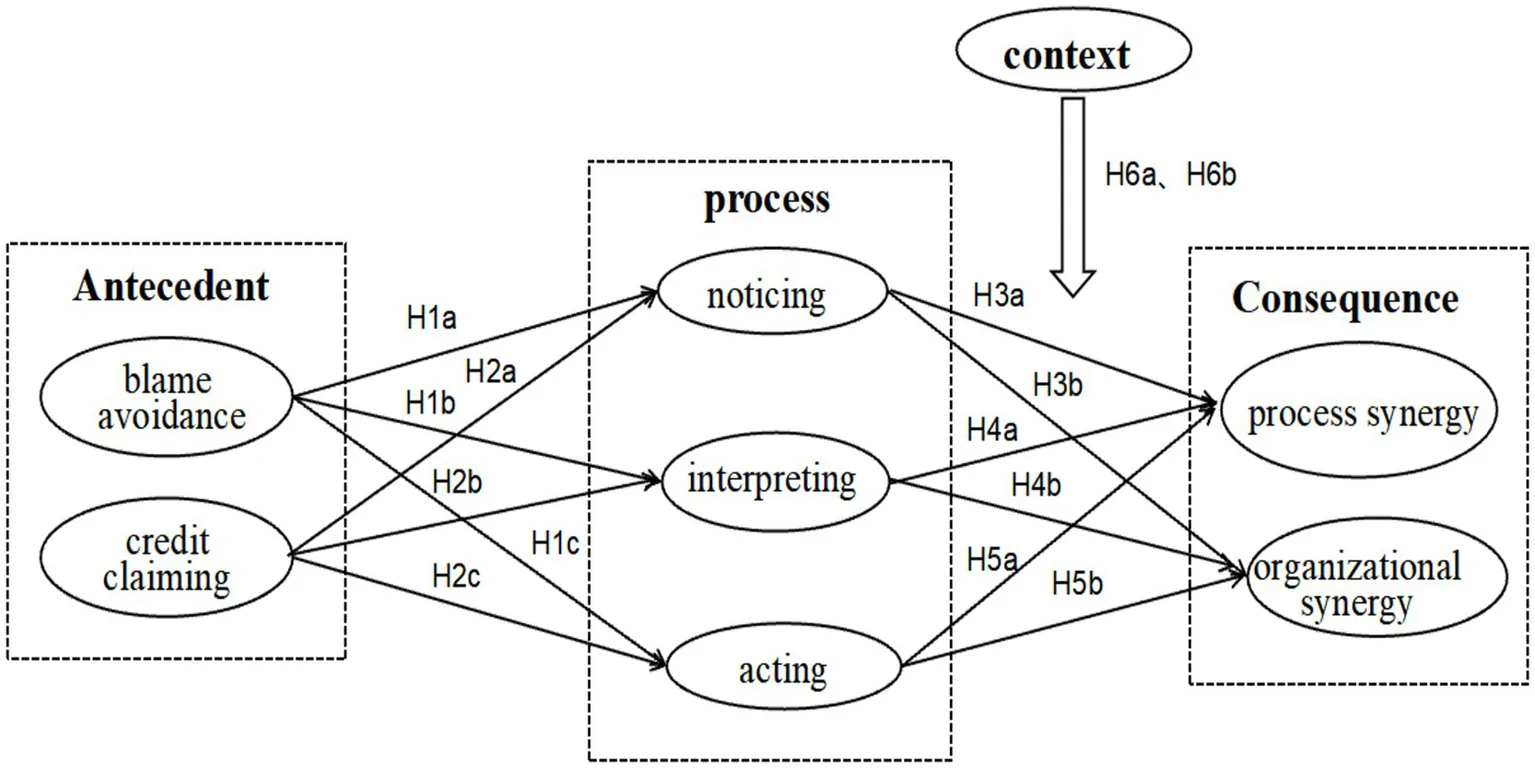
Theoretical model diagram.

## Methods

### Research design and data sources

This study adopts an exploratory sequential mixed-methods design to systematically examine the mechanism underlying the effect of leadership attention on online–offline healthcare service synergy. The qualitative phase integrates three data collection and analytical processes—semi-structured interviews, policy document analysis, and systematic literature review—to refine conceptual dimensions, contextualize core constructs, and generate an initial item pool; the quantitative phase collects data through a large-sample questionnaire survey and employs structural equation modeling as the primary analytical approach to test the hypothesized theoretical relationships and estimate path coefficients among latent variables. The two phases are tightly integrated via the logical pathway of “construct refinement–scale development–hypothesis testing”, ensuring that the measurement instruments align with both theoretical foundations and the practical context of healthcare management in China. Each of these three qualitative data collection and analytical processes is detailed in turn below.

#### Semi-structured interviews

To ensure the scale aligns with the practical context of healthcare management in China, participant recruitment strictly adheres to the following principles. (1) Relevance of job functions: Interviewees include leaders directly involved in medical decision-making and non-leader staff familiar with online-offline healthcare services, specifically 4 hospital directors of tertiary class A hospitals, 19 department directors, 8 section chiefs from the Health Commission, 2 research fellows, and 8 attending physicians. (2) Richness of medical practice experience: Participants are required to have medical management or clinical work experience and possess knowledge of online-offline service synergy. (3) Activeness in technology engagement: Priority is given to young and middle-aged leaders with high acceptance of digital technologies. The sample is predominantly aged 30–49 (35 participants), with supplementary representation from those under 30 (4 participants) and over 50 (2 participants). (4) High educational attainment: Most participants have received higher education, including 15 with bachelor’s degrees and 26 with postgraduate degrees. The interview guide was developed based on literature review and practical practices of online-offline healthcare service synergy, covering three core domains: perceivable leadership attention behaviors in synergy initiatives, motivational drivers of leaders’ attention allocation, and contextual conditions affecting synergy effectiveness.

To ensure the theoretical rigor and policy alignment of the questionnaire, this study concurrently conducted policy document analysis and literature analysis. At the policy document level, core policies on “Internet Plus Healthcare” issued at the national and provincial levels from 2018 to 2025 were selected to extract official formulations of leadership attention and healthcare synergy in policy discourse, so as to guarantee that questionnaire items conform to the industry policy context. At the literature level, a systematic scoping review was conducted to inform the conceptualization and measurement item development of the core constructs. Relevant studies published between January 2005 and March 2025 were retrieved from two electronic databases: China National Knowledge Infrastructure (CNKI) and Web of Science. Chinese-language publications were restricted to journals indexed in the Chinese Social Sciences Citation Index (CSSCI) and Chinese core journals, while English-language publications were limited to SCI/SSCI-indexed journals. For the theme of online–offline healthcare service synergy, search terms for Chinese records included “online-offline healthcare,” “O2O healthcare,” “internet healthcare,” “online healthcare” and “offline healthcare,” while terms for English records included “online and offline medical collaboration”, “O2O”, “internet medical,” “online medical” and “offline medical.” For the theme of leadership attention, search terms for Chinese records included “leadership attention” and “leader emphasis,” and “leadership attention” for English records. After deduplication, all retrieved records went through a two-stage screening process: initial relevance screening via titles, abstracts and keywords, followed by full-text validation. Finally, 212 valid articles (91 in Chinese, 121 in English) were retained for the online–offline healthcare synergy theme, and 215 (101 in Chinese, 114 in English) for the leadership attention theme.

ROST CM6 software was utilized for word frequency and thematic analysis to extract key terms associated with leadership attention allocation and the functioning of online-offline healthcare service synergy. An expert panel (comprising 1 professor of healthcare service management and 2 scientific researchers in the same field) was assembled to conduct semantic clustering and standardization of the initially screened vocabulary, laying a solid conceptual foundation for subsequent questionnaire development.

### Questionnaire design

#### Antecedent module

This module aims to measure the core motivational antecedents driving the allocation of different types of leadership attention. The measurement items are developed based on the blame avoidance–credit claiming framework in public administration and health policy implementation (22, 31). This module covers two core types: blame avoidance and credit claiming. Specifically, blame avoidance is measured by indicators including institutional constraints, risk supervision, and responsibility avoidance. Credit claiming is measured by indicators including performance incentives, promotion assessment, and political achievement recognition. A total of 10 initial items are developed for this module.

#### Process module

This module aims to measure the allocation process of different types of attention among health leaders. The theoretical foundation derives from the Attention-Based View (2) and the organizational interpretation perspective (32). Consistent with the theoretical framework of this study, healthcare leadership attention is categorized into three types: Noticing, Interpreting, and Acting. Noticing is measured by indicators including policy information monitoring, healthcare trend tracking, and synergy agenda focusing. Interpreting is measured by indicators including synergy value interpretation, goal analysis, and challenge interpretation. Acting is measured by indicators including special fund allocation, staffing arrangement, and institutional adjustment. A total of 15 initial items are developed for this module.

#### Context module

This module aims to measure the contextual boundary conditions that moderate the relationships between different types of leadership attention and online-offline healthcare service synergy performance. The selection of measurement dimensions is guided by the environmental dependence theory (2) and adapted to the practical context of China’s healthcare system reform. This module includes five contextual dimensions: public demand intensity for integrated healthcare, advancement of medical technical infrastructure, medical staff acceptance of service model transformation, support from superior health policies, and competitiveness of the local healthcare market. These dimensions are measured by indicators of public demand, technical advancement, organizational culture, policy environment, and market competition. A total of 5 initial items are developed for this module.

#### Consequence module

This module aims to measure the performance outcomes generated by online-offline healthcare service synergy. The measurement framework is based on the theoretical classification of system synergy in synergetics (33) and adjusted according to the characteristics of healthcare service delivery. This module contains two dimensions: organizational synergy performance and process synergy performance. Organizational synergy performance is measured by indicators including cross-departmental collaboration, resource sharing, and barrier removal. Process synergy performance is measured by indicators including service process integration, medical data sharing, and online-offline referral. A total of 10 initial items are developed for this module.

To enhance the reliability and construct validity of the questionnaire, a pilot test was conducted with 34 participants with relevant professional backgrounds: 20 master’s students in health services management, 2 doctoral candidates, and 12 frontline practitioners and junior administrators with direct experience of integrated online–offline healthcare services. The pilot was designed to identify ambiguous wording, assess initial construct validity, and refine the scale. The initial item pool comprised 40 measurement items. Following the pilot test, adjustments were implemented: semantically unclear items were revised for precision; 6 items with factor loadings below the conventional 0.5 threshold (such as item 5 of the blame avoidance dimension and item 4 of the noticing dimension) were removed based on exploratory factor analysis results. The final formal questionnaire contains 34 core measurement items, each rated on a 7-point Likert scale (1 = strongly disagree, 7 = strongly agree).

### Data collection

This study was approved by the Ethics Committee of Wenzhou Medical University (Approval No. 2025043). Questionnaire distribution and data collection were conducted using “wenjuanxing,” a widely used online survey platform in China. The platform’s sample database encompasses a broad and diverse population, with over 6.2 million registered users, and has been employed in more than 2,000 domestic studies (34). To enhance sample validity, explicit screening criteria were established prior to questionnaire distribution: respondents were required to primarily rely on internet-based medical platforms for disease consultation and to have prior experience with online health consultation services; in addition, individuals in leadership positions were identified.

In the sampling design, this study used quota sampling method. Referring to the regional distribution of Internet users in the statistical report on the development of China’s Internet Network (35), the sample quota proportions of the eastern, central and western regions are set at 60, 20 and 20%, respectively. A total of 1,100 questionnaires were distributed across three study regions, with 660, 220, and 220 copies allocated to each region, respectively. Overall, 690 questionnaires were returned, yielding an overall response rate of 62.7%. After excluding invalid samples — defined as those with an excessively short completion time (<120 s), failure to pass attention check items, or logical inconsistencies in responses — 450 valid questionnaires were retained for formal data analysis. The valid response rate among returned samples was 65.2%. The research process strictly followed the ethical norms of scientific research, and did not collect any personally identifiable information. All data were only used for academic research purposes.

### Statistical analysis

All data analysis was conducted using IBM SPSS Statistics 27.0 and IBM AMOS 24.0. Firstly, descriptive statistics and reliability and validity tests are conducted, and the standard fitting index is used to evaluate the overall fit of the measurement model. The structural equation model was applied to test the correlation proposed in hypotheses H1a-H5b. Finally, hierarchical regression analysis was used in conjunction with SPSS PROCESS Macro 4.0 (model with 5,000 lead samples, 95% confidence interval) to test and validate the moderating effect (hypotheses H6a-H6b) (36).

## Results

### Descriptive statistics

The final analytical sample comprised 450 respondents with direct experience in using or managing integrated online–offline healthcare services. Table 1 summarises the demographic characteristics. The sample was predominantly female (58.6%, *n* = 264), with a concentration in the young-to-middle-aged cohort: respondents aged 18–30 and 31–40 years accounted for 47.7 and 44.3%, respectively, together representing 88.3% of the total; those aged over 40 constituted only 8.0%. Educational attainment was notably high—95.3% of participants held at least a bachelor’s degree (associate degree: 82.2%; master’s degree or above: 13.1%). Regarding occupational roles, 11.3% (*n* = 51) were healthcare industry leaders involved in decision-making related to online–offline service synergy, while the remaining 88.7% (*n* = 399) were non-leader practitioners or service users, yielding a leader-to-non-leader ratio of approximately 1:7. Regional distribution followed the quota sampling design and mirrored the broader geographic pattern of internet healthcare users in China: 59.6% (*n* = 268) from Eastern China, 22.2% (*n* = 100) from Central China, and 18.2% (*n* = 82) from Western China.

**Table 1:** Demographic results.

Project	Category	Sample	Proportion
Gender	Male	186	41.4%
	Female	264	58.6%
Age	18–30 years old	215	47.7%
	31–40 years old	199	44.3%
	41–50 years old	34	7.5%
	51 years old and above	2	0.5%
Education	High school and below	21	4.7%
	Associate’s degree	370	82.2%
	Master’s degree and above	59	13.1%
Position	Healthcare industry leaders	51	11.3%
	Non-leaders	399	88.7%
Region	Western region	82	18.2%
	Central region	100	22.2%
	Eastern region	268	59.6%

In this study, leadership attention was measured using a two-stage, dual-perspective mixed-methods design that integrates leader self-reports and subordinate perceptions to deliver a comprehensive assessment of the construct.

In the qualitative research phase, we conducted semi-structured interviews with 41 healthcare organization managers, collecting first-hand data on leaders’ daily attention allocation practices. From the interview transcripts, we systematically extracted typical behavioral indicators corresponding to the noticing, interpreting, and acting dimensions of leadership attention, including meeting agenda setting and resource allocation prioritization. All core behavioral indicators were derived directly from leaders’ real-world practices in healthcare settings, ensuring that subsequent questionnaire items were grounded in actual leadership behaviors rather than abstract or subjective conceptual assumptions.

In the quantitative research phase, we took non-leader frontline staff as the primary sample while also including a proportion of leader respondents, and adopted a dual-perspective measurement approach that combines both the behavior actors and behavior recipients. Leader respondents, accounting for 11.3% (*n* = 51) of the total sample, reported the actual state of their own attention allocation from the perspective of decision-makers. This perspective presents the overall consideration and deployment logic of leadership attention at the management level, which can compensate for the limitation that frontline employees can only observe external behaviors but cannot perceive the decision-making context behind the behaviors. The 11.3% proportion of leader respondents aligns with the natural personnel distribution pattern of the pyramidal organizational hierarchy. Decision-makers and managers naturally constitute only a small share of the total workforce (37). Large-sample empirical studies in healthcare management, both in China and internationally, have consistently shown that management respondents typically account for 10 to 20% of research samples (38), and the sample proportion in this study falls well within this established reasonable range. Meanwhile, as the majority of the sample, non-leader frontline staff evaluated leadership attention behaviors based on their work experience with their supervisors. This perspective reflects the actual implementation effect of leadership attention in frontline service delivery, and can offset the potential subjective bias that may exist in leaders’ self-narratives. A meta-analysis by Podsakoff et al. (39) identified robust and consistent criterion-related validity between follower-rated leader behaviors and core work outcomes, verifying that employee perceptions can reliably capture actual patterns of leadership practice. Together, the two perspectives cover the complete process of leadership attention from internal decision-making to external implementation, integrate the deployment intention at the management level with the practical perception at the frontline level, and avoid the one-sidedness caused by relying solely on a single group. This dual-group design enables a more comprehensive and accurate examination of leadership attention allocation.

Descriptive statistical analysis (mean, standard deviation) and normality test (skewness, kurtosis) were conducted for all latent variables in the study to verify the data distribution characteristics, with results presented in Table 2. The skewness values of all variables ranged from −0.24 to −1.21, and the kurtosis values ranged from −0.73 to 2.21, all falling within the acceptable parametric statistical tests (40). The results of the normality test indicated that the study data conformed to approximate normal distribution, which supported the use of structural equation modeling (SEM) and hierarchical regression analysis for subsequent hypothesis testing.

**Table 2:** Mean and normality values.

Variables	Mean	Median	SD	Skewness	Kurtosis
Noticing	5.58	5.60	0.84	−1.21	2.1
Interpreting	5.45	5.60	0.74	−0.86	0.92
Acting	5.68	5.80	0.76	−1.05	2.41
Blame avoidance	4.33	4.40	1.25	−0.24	−0.73
Credit claiming	5.54	5.60	0.84	−1.09	1.54
Organizational synergy	5.76	5.80	0.70	−1.02	1.88
Process synergy	5.97	6.00	0.71	−1.09	1.25
Contextual factors	5.78	5.80	0.72	−1.16	2.21

### Reliability and validity

We assessed reliability and validity using SPSS 27.0 and AMOS 24.0. The overall Cronbach’s alpha coefficient was 0.895, indicating satisfactory internal consistency. Convergent validity was assessed using average variance extracted (AVE), composite reliability (CR), and standardized factor loadings. All CR values were above 0.7, and all standardized factor loadings exceeded 0.5. Although some AVE values fell below the ideal threshold of 0.5, they remained above 0.4—a level widely accepted in empirical research when composite reliability is sufficiently high (41). Detailed results are presented in Table 3. Discriminant validity was examined using the heterotrait-monotrait (HTMT) ratio. If the HTMT value is <0.90, discriminant validity is established (42). The HTMT values across all pairs of latent constructs ranged from 0.16 to 0.89, all of which met the discriminant validity criterion. The full HTMT matrix is presented in Table 4.

**Table 3:** Factor standard loadings, Cronbach’s *α*, CR, and AVE values.

Latent variable	Observed variable	Standardized loadings	Cronbach’s *α*	CR	AVE
Blame avoidance	BlameAvoidance1	0.79	0.87	0.89	0.68
	BlameAvoidance2	0.82			
	BlameAvoidance3	0.86			
	BlameAvoidance4	0.83			
Credit claiming	Credit Claiming1	0.82	0.79	0.86	0.60
	Credit Claiming2	0.78			
	Credit Claiming3	0.77			
	Credit Claiming4	0.72			
Noticing	Noticing1	0.61	0.78	0.74	0.42
	Noticing2	0.67			
	Noticing3	0.52			
	Noticing4	0.78			
Interpreting	Interpreting1	0.74	0.76	0.78	0.48
	Interpreting2	0.71			
	Interpreting3	0.72			
	Interpreting4	0.57			
Acting	Acting1	0.57	0.77	0.78	0.42
	Acting2	0.57			
	Acting3	0.67			
	Acting4	0.71			
	Acting5	0.62			
Process synergy	Process synergy1	0.75	0.68	0.77	0.41
	Process synergy2	0.69			
	Process synergy3	0.67			
	Process synergy4	0.55			
	Process synergy5	0.50			
Organizational synergy	Organizational synergy1	0.73	0.68	0.73	0.41
	Organizational synergy2	0.73			
	Organizational synergy3	0.59			
	Organizational synergy4	0.57			
	Organizational synergy5	0.55			
Contextual Factors	Contextual Factors1	0.88	0.64	0.77	0.54
	Contextual Factors2	0.77			
	Contextual Factors3	0.51			

**Table 4:** HTMT ratio.

Variables	Blame avoidance	Credit claiming	Noticing	Interpreting	Acting	Organizational synergy	Process synergy	Contextual factors
Blame avoidance								
Credit claiming	0.31							
Noticing	0.30	0.26						
Interpreting	0.33	0.29	0.89					
Acting	0.39	0.34	0.77	0.87				
Organizational synergy	0.27	0.33	0.55	0.63	0.70			
Process synergy	0.16	0.40	0.54	0.58	0.62	0.70		
Contextual factors	0.22	0.55	0.57	0.61	0.73	0.62	0.52	

HTMT, heterotrait-monotrait ratio. Values on the diagonal are omitted (equal to 1.0).

### Common method deviation detection

Harman single factor test was used for common method deviation test (43). The results show that there are 8 factors with characteristic roots >1. The variation explained by the first factor is 23.94%, which is less than the critical standard of 40%, indicating that there is no serious common method.

### Structural equation model analysis

The hypothesized measurement model was examined via structural equation modeling (SEM). After model modification, the fit indices of the revised model were as follows: *χ*^2^/df = 3.742, RMSEA = 0.078, CFI = 0.824, GFI = 0.867, AGFI = 0.867. Given the model complexity and sample size, such fit performance is considered acceptable (44).

Structural equation modeling was conducted using AMOS 24.0 to estimate the associations and effect directions among variables and test Hypotheses H1a to H5b. The standardized path coefficients and significance levels are presented in detail in Figure 2. The analyses focused on two core themes: the effect paths of different types of leadership attention on online-offline healthcare synergy performance, and the influencing mechanisms of motivational antecedents on the allocation of distinct forms of leadership attention.

**Figure 2:**
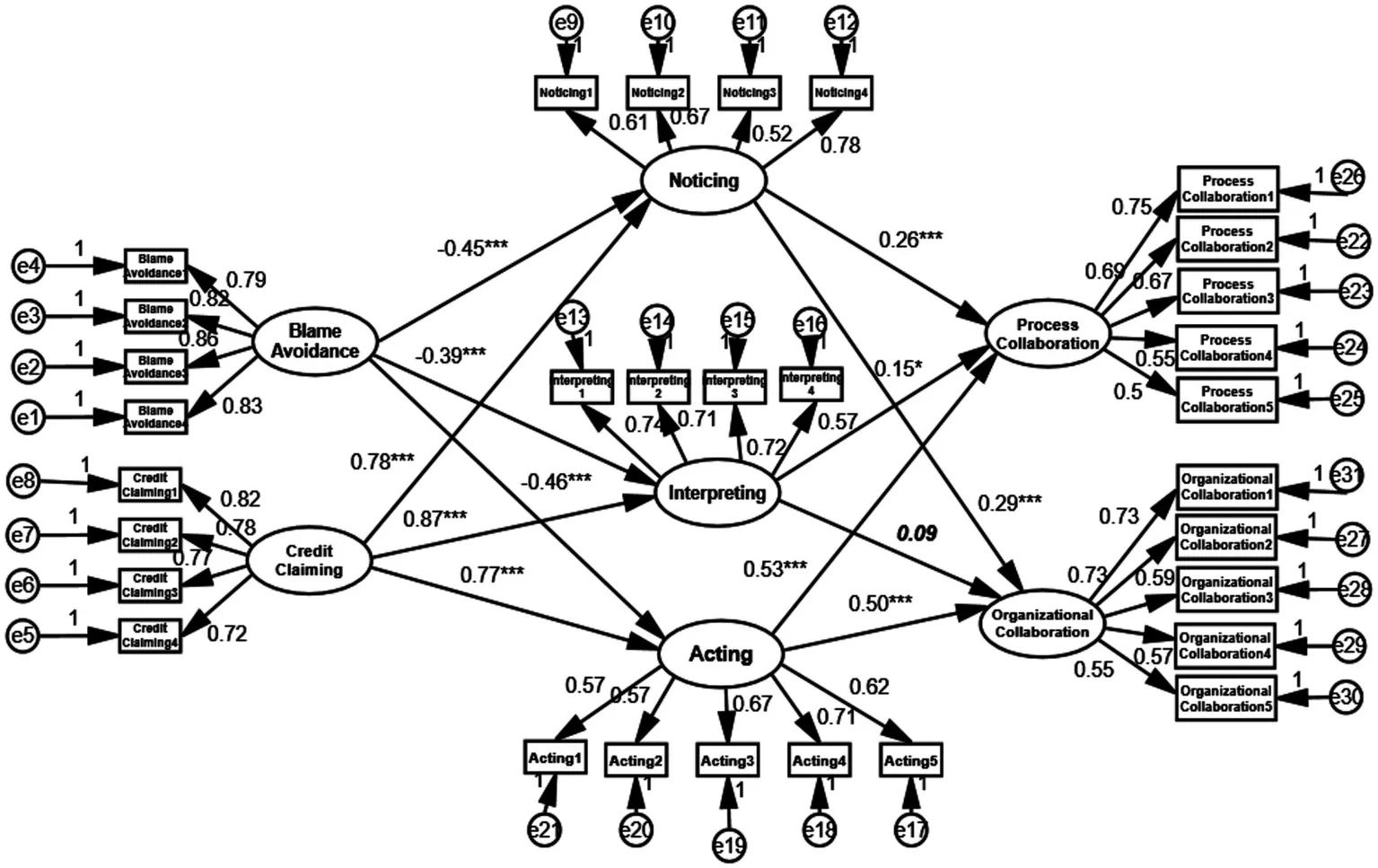
Standardized path coefficients of leadership attention on the synergy of online and offline healthcare services. ***p < 0.001, **p < 0.01, *p < 0.05.

This study first examined the effects of three types of leadership attention on synergy performance (H3a–H5b). All hypotheses were supported except for H4b. Noticing had significant positive effects on both types of synergy performance (process synergy: *β* = 0.26, *p* < 0.001; organizational synergy: *β* = 0.29, *p* < 0.001), supporting H3a and H3b. This aligns with the core proposition of the Attention-Based View that agenda attention affects the priority of resource investment. Interpreting significantly promoted process synergy performance (*β* = 0.15, *p* < 0.05; H4a supported), but showed no statistically significant effect on organizational synergy performance (*β* = 0.09, *p* > 0.05; H4b not supported). This finding indicates that leaders’ interpretation of collaborative goals and pathways can only effectively drive technical process integration and optimization, yet is insufficient to overcome deep structural barriers in organizational synergy such as interest redistribution and power restructuring. Acting showed the strongest positive association with both dimensions of synergy performance (process synergy: *β* = 0.53, *p* < 0.001; organizational synergy: *β* = 0.50, *p* < 0.001), supporting H5a and H5b. It indicates that translating leadership attention into substantive actions—including special fund allocation and inter-organizational institutional adjustment—is associated with addressing dilemmas in online-offline healthcare integration, which echoes the findings of Wang et al. (45) that leadership attention affects healthcare quality through resource mobilization.

Subsequent path analyses tested the associations of blame avoidance (H1a–H1c) and credit claiming (H2a–H2c) with the three forms of leadership attention. Blame avoidance was significantly negatively associated with Noticing (*β* = −0.45, *p* < 0.001), Interpreting (*β* = −0.39, *p* < 0.001), and Acting (*β* = −0.46, *p* < 0.001), with the strongest negative association observed for Acting. Thus, H1a, H1b, and H1c were fully supported. Credit claiming displayed significant positive effects on all three types of attention (Noticing: *β* = 0.78, *p* < 0.001; Interpreting: *β* = 0.87, *p* < 0.001; Acting: *β* = 0.77, *p* < 0.001), especially on Interpreting, supporting H2a, H2b, and H2c. Further comparative analyses revealed that the absolute path coefficient of credit claiming was substantially larger than that of blame avoidance. This result indicates that within China’s pressure-oriented healthcare administration system, credit claiming is more strongly positively associated with leaders’ attention allocation toward online-offline integration than blame avoidance is negatively associated with it, which is consistent with research on leadership behavioral motives in public administration (46).

### Analysis of moderation effect

Hierarchical regression analysis and the PROCESS 4.0 macro were used to test the moderating effects of contextual factors. To mitigate multicollinearity, mean centering was performed on the independent variables (aggregated types of leadership attention) and moderating variables (contextual factors). The interaction term between leadership attention and contextual factors had no significant effect on process synergy performance (*β* = 0.054, *p* > 0.05), thus Hypothesis H6a was not supported. However, this interaction term exerted a significant positive effect on organizational synergy performance (*β* = 0.113, *p* < 0.01), supporting Hypothesis H6b. This finding indicates that contextual factors do not moderate the relationship between leadership attention and process synergy performance, but can significantly enhance the positive effect of leadership attention on organizational-level synergy performance. This is consistent with the core proposition of the Attention-Based View that “contextual fit affects the effectiveness of attention” (2). Process synergy performance relies more on the technical connection and standardized operation of service processes, and is less affected by external contextual factors. In contrast, organizational synergy performance involves in-depth issues such as cross-organizational responsibility restructuring and interest coordination. Favorable contextual conditions can provide necessary guarantees for the transformation of leadership attention, thereby amplifying its driving effect on organizational synergy. Detailed results are presented in Table 5.

**Table 5:** Moderating effects in second-order regression analysis.

Variable		Process synergy				Organizational synergy			
		S1	S2	S3	S4	S5	S6	S7	S8
Control variable	Gender	0.009	0.01	0.001	0.007	0.007	0.008	−0.008	0.005
	Age	0.092	0.007	0.008	0.007	0.104	0.012	0.013	0.011
	Education level	−0.029	−0.096	−0.099	−0.095	0.035	−0.038	−0.043	−0.036
Independent variable	Noticing		0.483^***^	0.414^***^	0.425^***^		0.526^***^	0.397^***^	0.421^***^
Moderator variable	Contextual factors			0.127	0.137			0.236^***^	0.257^***^
Interaction item	Noticing × contextual factors				0.054				0.113^**^
Model summary	*R* ^2^	0.017	0.244	0.255	0.257	0.014	0.283	0.322	0.333
	Adjusted *R*^2^	0.005^**^	0.275	0.313	0.322	0.008^**^	0.235	0.245	0.245
	*F*	1.607	34.953^**^	34.961^**^	31.408^**^	1.928	28.519^***^	25.191^***^	21.823^***^

***p < 0.001, **p < 0.01, *p < 0.05.

To further verify the robustness of the aforementioned moderating effect test results, this study examined the moderating effects of contextual factors using the PROCESS macro version 4.0 with Model 1 (simple moderation model). Bootstrapping was conducted with 5,000 resamples and a 95% confidence interval.

When organizational synergy was specified as the dependent variable, the interaction term between the interpreting dimension of leadership attention and contextual factors returned a *p*-value of 0.01 (*p* < 0.05), with a 95% confidence interval of [0.02, 0.14] that excluded zero, indicating a statistically significant moderating effect. Both the coefficient of leadership attention and the interaction term coefficient were positive, demonstrating that contextual factors positively moderate the relationship between the interpreting dimension and organizational synergy. This finding provides further empirical support for Hypothesis H6b.

When process synergy was specified as the dependent variable, the interaction term between leadership attention and contextual factors had a *p*-value of 0.13 (*p* > 0.05), with a 95% confidence interval of [−0.01, 0.11] that included zero. The moderating effect was therefore not statistically significant, confirming that Hypothesis H6b is not supported. The results of the PROCESS-based moderation test are fully consistent with those derived from hierarchical regression analysis. Detailed results are presented in Tables 6.

**Table 6:** Process adjustment effect test.

Category	Process synergy				Organizational synergy			
	Coefficient	Standard error	95%CI	*p*	Coefficient	Standard error	95%CI	*p*
Attention	0.41	0.05	[0.32,0.50]	0.00	0.39	0.04	[0.31,0.49]	0.00
Situation	0.11	0.04	[0.03,0.19]	0.01	0.20	0.04	[0.13,0.28]	0.00
Attention × situation	0.49	0.01	[−0.01,0.11]	0.13	0.08	0.03	[0.02,0.14]	0.01
*R* ^2^	0.004				0.011			

## Discussion

This study’s core findings indicate that different types of leadership attention are associated with online-offline healthcare synergy performance in differentiated patterns. Among the three types, Interpreting has a weaker association than Noticing, while Acting exhibits the strongest positive and statistically significant association with synergy performance improvement. This result highlights the priority of substantive actions over symbolic attention and cognitive interpretation in online-offline healthcare synergy, which aligns with the practical superiority of “substantive governance” over “symbolic governance” in public health policy implementation (16).

Within China’s hierarchical healthcare system, Noticing performs a foundational agenda-setting function by issuing policy signals and consolidating cross-organizational cognitive consensus, but it remains largely a symbolic commitment. Without corresponding resource input and institutional guarantees, it can easily fall into the dilemma of “performative synergy” (27). Interpreting further translates broad policy agendas into explicit strategic goals, implementation pathways and value orientations through organizational sense-making. For process synergy—an operational-level outcome centered on service chain optimization, workflow standardization and data interoperability—such interpretive work effectively reduces operational ambiguity and aligns frontline expectations, thus facilitating seamless integration across service segments. However, this cognitive framing effect does not extend to organizational synergy, which requires restructuring cross-departmental authority, redistributing interests and reforming institutional arrangements. Qualitative evidence from interviews corroborates this limitation: as one department director from a tertiary hospital stated, “We have held dozens of coordination meetings to interpret the value and roadmap of online-offline integration, but when it comes to adjusting staffing quotas, merging administrative functions or reallocating performance bonuses across departments, verbal emphasis and goal clarification alone cannot break through long-established departmental boundaries.” This bureaucratic friction explains why interpretive attention fails to drive organizational-level synergy (47): it addresses cognitive alignment but cannot resolve the structural incentives and power barriers inherent in public hospital governance (48).

In contrast, Acting converts attentional focus into substantive, resource-embedded measures—including dedicated fund allocation, staffing restructuring, performance assessment reform and unified service standards—which directly reshape stakeholders’ interest calculations and risk perceptions. It serves as the critical link that transforms cognitive synergy consensus into practical outcomes. A health bureau official interviewed further noted, “Real integration only advances when we tie synergy performance to annual evaluation and allocate special budgets; otherwise, it stays on documents and meeting minutes.” This confirms that in the Chinese context, driving substantive synergy requires leadership attention to shift from “signal release” to “resource commitment” (49).

Notably, the associations of leadership attention with different dimensions of synergy performance are heterogeneous. For organizational synergy performance, only Noticing and Acting exert significant driving effects (Interpreting has no statistical significance); for process synergy performance, all three types of attention show positive associations, with the strongest association observed for Acting. This finding enriches the contingency perspective of leadership attention research, and the differences stem from the distinct intrinsic attributes of the two types of synergy performance: the improvement of organizational synergy performance relies on interest adjustment and institutional reform. Mere “Interpreting” cannot surmount the existing structural barriers in public hospital reform; only top-down institutional design and resource allocation can take effect. In contrast, the improvement of process synergy performance focuses on technical coordination: Noticing can unify cognitive expectations, Interpreting can clarify operational norms to reduce implementation ambiguity, and together with the resource guarantee of Acting, they form a joint force.

Therefore, leaders need to adopt differentiated strategies: for in-depth organizational synergy, priority should be given to the input of Acting-type attention; for technical process synergy, all three types of attention can be comprehensively applied. A “one-size-fits-all” strategy that ignores the attributes of synergy performance will lead to misalignment of attention allocation, thereby undermining the effectiveness of synergy performance improvement.

Blame avoidance and credit claiming show asymmetric associations with leadership attention allocation. Both motives significantly influence the three types of leadership attention, but the driving intensity of credit claiming is much higher than the inhibitory effect of Blame Avoidance, with obvious differences in their action paths: Blame Avoidance and credit claiming show asymmetric associations with leadership attention allocation, while credit claiming shows the strongest positive association with Interpreting (50). This finding enriches the application scenario of the blame avoidance–credit claiming theory in the field of leadership and reveals the micro-political logic of leadership attention allocation under the pressure-oriented system. The asymmetric characteristics originate from the incentive structure and accountability mechanism under China’s pressure-oriented system. Policy implementation effectiveness has become a key indicator for officials’ promotion assessment, driving leaders to actively mobilize attention resources to create visible performance highlights, thus making credit claiming dominant. Although blame avoidance prompts leaders to adopt defensive strategies to avoid accountability risks, its inhibitory effect is relatively limited under the dual influence of top-down policy promotion and performance-oriented incentive mechanisms (51). This asymmetry leads to a “visibility bias” in attention allocation: leaders tend to prioritize resource allocation to collaborative links that are easy to quantify and highly visible, while avoiding areas with high potential risks and slow results. This also explains why online-offline healthcare synergy has made rapid progress in the technical process integration level, but has advanced slowly in the organizational synergy level involving institutional restructuring.

Contextual factors exert differentiated moderating effects on the relationship between leadership attention and synergy performance. Contextual factors positively moderate the relationship between leadership attention and organizational synergy performance, but have no significant moderating effect on the relationship between attention and process synergy performance. This finding clarifies the boundary conditions for leadership attention to drive synergy performance and reveals the differences in the dependence of the two types of synergy performance on the external environment. The improvement of organizational synergy performance is essentially an institutional reform, which is closely associated with a favorable macro environment—mature medical technology infrastructure can provide technical support, clear superior policy support can enhance the legitimacy of synergy, and strong public demand can reduce cross-organizational coordination costs. These factors jointly strengthen the positive association between leadership attention and organizational synergy; unfavorable environmental conditions, however, will severely restrict the effectiveness of leadership attention. The improvement of process synergy performance relies more on the internal operational capabilities of medical institutions (such as the technical level of front-line personnel and project management capabilities), and external contextual factors only provide basic background support, with relatively limited moderating effects. This means that optimizing macro contextual conditions (such as strengthening policy support and improving technical infrastructure) is an important prerequisite for enabling leadership attention to drive the improvement of organizational synergy performance; while the improvement of process synergy performance should focus on internal capacity building (such as technical training and management process optimization) rather than over-reliance on external environmental factors.

## Conclusion

Based on the integrative Antecedent-Process-Context-Consequence (APCC) framework, this study constructed and tested a theoretical model of how leadership attention allocation influences online-offline healthcare service synergy performance. The study revealed the differentiated associations of three types of leadership attention (Noticing, Interpreting, and Acting) with synergy performance, clarified the asymmetric driving logic of blame avoidance and credit claiming in shaping attention allocation, and identified the moderating boundaries of contextual factors, providing a systematic perspective for understanding leadership behavior in policy implementation in the healthcare field.

### Theoretical implications

This study makes three main theoretical contributions to the fields of leadership, health policy implementation, and online-offline healthcare integration research:

First, it fills the perspective gap in online-offline healthcare synergy research. Existing studies mostly focus on process optimization, resource allocation, and technology empowerment to explore online-offline healthcare synergy, but have not analyzed its influencing mechanism from the perspective of leadership attention. This study decomposes leadership attention into three independent types (Noticing, Interpreting, and Acting), empirically verifies the differentiated associations of each type with synergy performance, enriches the application scenario of the Attention-Based View in the healthcare field, and provides a new theoretical perspective for understanding the internal mechanism of leadership behavior driving digital health transformation.

Second, it integrates the blame avoidance–credit claiming theory and attention theory to improve the antecedent research on leadership attention allocation. This study empirically tests the asymmetric driving mechanism of blame avoidance and credit claiming on different types of leadership attention, reveals the micro-political logic of attention allocation under China’s pressure-driven healthcare administration system, supplements the research on the interaction between leadership motivation and attention in the field of public administration, and provides a new analytical framework for interpreting the motivational roots of leadership behavior.

Third, it clarifies the contingent conditions for leadership attention to influence synergy performance, enriching the empirical basis of contingency leadership theory. This study identifies the contingent effect of synergy performance attributes and the differentiated moderating role of contextual factors, confirms that the effectiveness of leadership attention depends on the adaptation between synergy performance types and the external environment, expands the contingency perspective of leadership attention research, and provides empirical support from the Chinese context for the deepening of contingency leadership theory in the healthcare field.

### Practical implications

This study provides targeted practical insights for health administrative departments and medical institutions to optimize leadership attention allocation and promote the in-depth integration of online-offline healthcare services:

First, promote the transformation of leadership attention from symbolic expression to substantive action. Health administrative departments should establish an assessment mechanism oriented to synergy performance, guide leaders to increase investment in Acting-type attention, and through specific measures such as special fund allocation and adjustment of inter-organizational collaboration systems, avoid “performative attention” that only stays at agenda setting and sense-making, ensuring that attention resources are truly transformed into synergy effectiveness.

Second, optimize the incentive and accountability structure to balance the impact of the two types of motives on attention allocation. On the one hand, link the effectiveness of online-offline healthcare integration with performance evaluation and promotion, strengthening the positive driving role of credit claiming; on the other hand, establish a scientific fault-tolerance mechanism to reduce the accountability risks of leaders in synergy reform, avoid insufficient investment in Acting-type attention due to excessive blame avoidance, and form a motivation guidance system with “effective incentives and appropriate fault tolerance”.

Third, implement differentiated leadership attention allocation strategies according to the attributes of synergy performance. For the improvement of organizational-level synergy performance involving institutional reform and interest adjustment, focus on strengthening the investment of Acting-type leadership attention; for the improvement of operational-level synergy performance focusing on technical coordination and process optimization, comprehensively use the three types of attention (Noticing, Interpreting, and Acting) to form a joint force through unifying cognition, clarifying norms, and ensuring resources.

### Study limitations and future research directions

First, the cross-sectional research design can only reveal variable correlations rather than causal inferences, and cannot capture the dynamic evolution of leadership attention and synergy performance across implementation stages. Second, this study uses subordinates’ perceptions to measure leadership attention. Despite anchoring items to observable behaviors derived from prior qualitative interviews, individual perceptual bias cannot be fully eliminated, and the small share of leader respondents prevents analysis of how leaders’ personal traits are associated with attention allocation. Third, the findings are rooted in China’s centralized bureaucratic healthcare governance, so generalizability to healthcare systems with different administrative frameworks is limited.

Future research can be advanced in three corresponding directions: (1) Adopt longitudinal tracking and collect panel data to explore causal dynamics between leadership attention and synergy performance; (2) collect matched leader-subordinate paired data to improve measurement validity and examine leaders’ trait associations with attention allocation; (3) conduct cross-national comparisons to verify the robustness of results under alternative healthcare governance systems.

## Data Availability

The original contributions presented in the study are included in the article/Supplementary material, further inquiries can be directed to the corresponding author.
