# Supplementary material for: How leadership attention is associated with online–offline healthcare service synergy: differentiated effects of noticing, interpreting, and acting
Source: Front Public Health. 2026 Sep 29;14:1956639. doi: 10.3389/fpubh.2026.1956639 (PMC13658531; doi:10.3389/fpubh.2026.1956639)
Supplement: Supplementary file 1 [file Table_1.DOCX]

**Table A1. Full version questionnaire**

| **Category** | **Measurement Item** | **Source** |
| --- | --- | --- |
| Blame Avoidance | In promoting the integration of online and offline healthcare services, leaders tend to prioritize simple tasks while avoiding complex issues. | Based on Hood [23], Xu Ke [24], WeaverR.K.[12] |
|  | In promoting the integration of online and offline healthcare services, leaders tend to strictly adhere to established procedures. |  |
|  | In promoting the integration of online and offline healthcare services, leaders tend to avoid taking risks by sticking to the status quo rather than trying new approaches. |  |
|  | In promoting the integration of online and offline healthcare services, leaders tend to report only successes to superiors while concealing difficulties. |  |
|  | In promoting the integration of online and offline healthcare services, leaders tend to attribute integration challenges to external factors. |  |
| Credit Claiming | In driving the integration of online and offline healthcare services, leaders prioritize whether integration performance serves as a key metric for job promotions. | Based on Hood [23] , Xu Ke [24], Weaver R.K.[12] |
|  | In driving the integration of online and offline healthcare services, leaders prioritize whether integration performance is linked to compensation incentives. |  |
|  | When promoting the integration of online and offline healthcare services, leaders prioritize whether collaborative performance gains upper management recognition. |  |
|  | When promoting the integration of online and offline healthcare services, leaders prioritize whether collaborative performance relates to honors such as “Outstanding Worker.” |  |
|  | When promoting the integration of online and offline healthcare services, leaders emphasize proactively publicizing collaborative achievements. |  |
| Noticing | Leaders monitor multiple channels for obtaining information on the integration of online and offline healthcare services. | Based on Ocasio [8]and Daft and Weick [25] |
|  | Leaders focus on how to filter information regarding the integration of online and offline healthcare services. |  |
|  | Leaders monitor policies related to online-offline healthcare service integration. |  |
|  | Leaders track patient feedback on online-offline healthcare service integration. |  |
|  | Leaders observe technological trends in online-offline healthcare service integration. |  |
| Interpreting | Leaders maintain a clear understanding of integration objectives. | Based on Ocasio [8]and Daft and Weick [25] |
|  | Leaders conduct in-depth analyses of integration challenges. |  |
|  | Leaders can articulate the strategic value of online-offline healthcare service integration. |  |
|  | Leaders can refine integration plans through collaborative discussions. |  |
|  | Leaders fully consider the long-term benefits of online-offline healthcare service integration. |  |
| Acting | Leaders allocate specialized personnel to support the development of online-offline healthcare service integration. | Based on Ocasio [8]and Daft and Weick [25] |
|  | Leaders introduce advanced technologies to support the development of online-offline healthcare service integration. |  |
|  | Leaders ensure financial investment to support the development of online-offline healthcare service integration. |  |
|  | Leaders dynamically adjust collaborative work methods to support the development of online-offline healthcare service integration. |  |
|  | Leaders enhance collaborative capabilities through regular training to support the development of online-offline healthcare service integration. |  |
| Contextual Factors | Public demand influences leaders' prioritization of online-offline healthcare service integration. | Based on Ocasio [8] |
|  | Policy environment influences leaders' prioritization of online-offline healthcare service integration. |  |
|  | Employee recognition influences leaders' prioritization of online-offline healthcare service integration. |  |
|  | Technological advancement influences leaders' prioritization of online-offline healthcare service integration. |  |
|  | Market competition intensity affects leaders' emphasis on online-offline healthcare service coordination. |  |
| Process synergy | Online-offline healthcare service coordination enables seamless integration throughout the entire patient care journey. | Based on Haken [16] |
|  | Online-offline healthcare service coordination facilitates smooth online-to-offline referrals. |  |
|  | Online-offline healthcare service coordination enables cross-institutional medical record data sharing. |  |
|  | Online-offline healthcare service coordination achieves mutual recognition of patient test results across online and offline platforms. |  |
|  | Online-offline healthcare service coordination implements consistent quality control standards. |  |
| Organizational synergy | Online-offline healthcare service integration requires establishing cross-departmental leadership teams. | Based on Haken [16] |
|  | Online-offline healthcare service integration requires establishing clear systems for delineating authority and responsibility. |  |
|  | Online-offline healthcare service integration requires establishing cross-organizational communication mechanisms. |  |
|  | Online-offline healthcare service integration requires establishing unified information management systems. |  |
|  | Online-offline healthcare service integration requires establishing unified on-call systems. |  |
